# Abstracts and Gleanings

**Published:** 1879-05-20

**Authors:** 


### ABSTRACTS AND GLEANINGS.

**Prof. Jones' Experiments with the Water and Air from Yellow Fever Rooms.**—In a late number of the New Orleans Medical Journal, Prof. Jones presents an interesting paper on yellow fever, from which we extract the following:

When the water from the yellow fever rooms was subjected to microscopical examination, the following extraneous matters were observed:

1st. Numerous minute particles, many of which had a vibratory motion. Under a magnifying power of 420 diameters, with Beck's best 1-15th of an inch (a superior glass of excellent defining power), these appeared as minute oval specks. Under 1-18th of an inch (1050 diameters) these particles were resolved into distinct oval cells with a central nucleus, resembling in all respects the spores of delicate fungi.

2d. Bacteria and delicate thread-like filaments, similar to those observed in the urine and in the blood of yellow fever.

3d. Revolving minute animalculæ and spores, with active rotatory movements.

4th. Minute particles which could not be resolved into distinct structures by the highest powers. When magnified 1050 diameters, these resembled mere specks of matter, many of which have an active vibratory motion.

5th. Epithelial cells.

6th. Particles of dust evidently inorganic in their nature.

7th. Oil globules. As the patients were well rubbed with olive oil the oil globules may have in part been derived from this source.

8th. Hairs and particles of cotton and sheep wool from the clothing and bedding of the patients with numerous adherent spores.

When the liquid from the yellow fever rooms was evaporated, a distinct deposit was left in the watch glasses and upon glass slides, which, in addition to the various organic substances specified, contained numerous stellate and accicular and prismatic crystals and granular particles. The crystals appear to be those of the chloride and carbonate of ammonia. Reaction of water slightly alkaline.

The presence of organic matter was still farther shown by the usual chemical tests, as charring by heat, blackening by sulphuric acid, and decoloration of the solution of the permanganate of potassa.

When glass slides were moistened with ice cold water and held so as to receive the breath of yellow fever patients in respiration, the microscopical examination yielded results similar to those recorded above.

After the most minute examination of the individual specimens from the different rooms in Mr. Harrison's house, not only immediately after the experiments, but also during various periods, embracing nearly six months, I discovered no forms which could be referred to such microscopical plants as the chlorococcum vulgare, protococcus viridis, palmella cruenta, coccochloris brebinonii and other confervoideæ, or unicellular algæ capable of producing chlorophyl. Certain granular cells observed in malarial fever (in the blood), resemble most nearly the resting spore of bulbochæte intermedia, and the granular cells of palmella cruenta; but no such cells were observed in the yellow fever atmosphere in the brick house 363 Magazine street. In fact it would be difficult to conceive how the algæ of any description could thrive and multiply in this well paved and dry situation. The forms were referable to those most nearly connected with putrefaction and fermentation, as the bacteria and torulæ, penicillius and micrococci and cryptococceæ. Kutzing includes his genera cryptococcus, ulvina and sphærotilus amongst the family of algæ, but they appeared to be the conidia (reproduction cells, stylospores and spermatia) of the mycelia of mildew fungi. The absence of any of the known forms of the algæ in the air of yellow fever collected in this locality, which is as free from any source of swamp malaria as the best drained and paved portions of the city of New Orleans, is important in that this class of plants is thus excluded from the consideration of the questions relating to the origin and causation of yellow fever.

The air from the sick rooms presented the same elements as were observed in the preceding experiments on Magazine street, with the addition of several colored cells which evidently belonged to the plants, resembling the chlorococcum vulgare, protococcus viridis and palmella cruenta. The true characters of these elements were fully shown by examinations of the water, at various points extending over five months, during which time the cells increased considerably in size. The most numerous elements were as in the case of the water obtained from the sick room at 363 Magazine street, minute spores, minute threadlike bodies, bacteria and minute granules which could not be resolved by the highest powers into any distinct animal or vegetable structure; epithelial cells, oil globules, several parasites from the skin, and fibres of wool and cotton, and particles of dust were also observed under the microscope. The crystalline bodies also appeared when the liquid was slowly coagulated.

The water through which the air over the foul gutter was passed contained chiefly cells of the plants resembling the algæ, as the testing spores of bulbochæte intermedia, chlorococcum vulgare, protococcus viridis and palmella cruenta. Bacteria and minute spores and minute particles were also abundant.

When the waters obtained in the manner stated from this locality were injected subcutaneously into living animals, the results were similar to those previously detailed.

**Morphia Hypodermically—Caution.**—Ocasional fatal results have been reported from the use of morphine subcutaneously. Such cases may be due to idiosyncrasy, or more probably to the careless use of the drug. It should be borne in mind that the power of a remedy is increased threefold by the hypodermic method of medication. Upon this subject the following hints are from remarks of Dr. Gibbons in San Francisco Medical Society, reported in *Pacific Med. Journal.*

1. Avoid it in congestion and inflammatory conditions of the brain.
2. Avoid it in pulmonary congestion, and where dyspnea is not the result of spasm.
3. Avoid it in acute inflammatory affections of the heart and pericardium.
4. Avoid it in high febrile excitement.
5. Avoid puncturing a vein. The effect of introducing morphia into a vein is instantaneous. It is possible that the evil results were due in some cases to the entrance of the narcotic into a small vein—so small and deep as to escape observation.

6. Avoid a deep puncture, unless there is a special purpose to be accomplished by depositing the narcotic deep in the tissues.

7. Introduce the liquid slowly, and not by sudden projection, as is sometimes done. Spend four or five seconds in driving the piston home.

8. Require the patient to lie down and remain quiet after the operation. There is some risk in moving about, independently of the nausea which follows. The syringe should be used in the patient's chamber, and not in the doctor's office.

I may add, in regard to the appliance in cardiac cases, that according to my experience, there is no condition of human suffering to which it is more applicable than in the agonizing orthopnea of cardiac asthma. In fact, it is the remedy, par excellence, for the paroxysm of spasmodic asthma, from whatever cause.

By observing the foregoing cautions, I think the risk of injury from the hypodermic use of morphia will be reduced to a minimum, whilst the physician will be armed with an instrumentality for the relief of pain, the value of which is beyond calculation.

A singular phenomenon, which I am not able to explain satisfactorily, has come under my observation in some exceptional cases, namely the aggravation of pain immediately after the injection. The patient complains that the pain is suddenly augmented, say in twenty or thirty seconds after the injection; but the aggravation is brief, passing away in less than a minute, and is succeeded by the proper action of the drug.

Another effect is much more common—a distressing sensation which takes the place of the pain, and causes the patient to complain that he, or more frequently she, feels as if dying. "I feel as if I was dying," is the expression. It is the stepping-stone, so to speak, between pain and hypnotism. It lasts only a fraction of a minute, and vanishes with the accession of the hypnotic stage. I confess to a feeling of anxiety whenever it occurs, and am careful to require the patient to keep quiet in the horizontal posture.

**Female Catheterism.**—Dr. Millikin (Ob. Gazette) says:

Some months ago I was asked to see a woman who had gone through a long and violent labor under the care of a midwife, and who had in her bladder fifty hours' accumulation of urine. To my chagrin I found myself quite unable to recognize the meatus urinarius by the sense of touch. I happened to have a long gum catheter and a small glass speculum in my satchel, and the "happy thought" occurred to me that I might avoid exposing her by catheterizing her through the speculum. I caused the speculum to fairly enter the vagina and then, gently withdrawing it, I was able to see, what no man can feel, that the meatus was thrown far to the left side, and could only be reached by passing the point of the catheter along a furrow, which was partly formed and partly concealed by an overhanging fold of sloughing mucous membrane. Aided by the excellent illumination I was able to disinfect the region quickly and to empty the bladder.

I have recently made use of the same simple apparatus in catheterizing a puerperal patient who had an astonishing degree of tenderness and irritability about the ostium vaginæ, and I do not think the operation was ever performed more quickly and comfortably.

It seems to me that it would be well to resort to the speculum to separate the labia and illuminate the region between them whenever there is difficulty in discovering the exact site of the meatus. Even when they succeed, prolonged efforts to insert the catheter by plowing over the whole vestibule are sure to cause pain and loss of confidence on the part of the patient; while in case of failure, there is nothing more mortifying to patient and physician than the common mode of ocular investigation.

**The Application of Cold and the Elevation of an Extremity as Hæmostatics.**—It appears from recent researches, that as prophylactic against hemorrhage, Esmarch's bandage does not answer all the requirements demanded of it by over-exacting critics, who have found it necessary to unearth and resuscitate antiquated practices, to serve as happy illustrations of the practical application of results experimentally obtained. In this regard the recent (?) researches of Dr. Wolff (*Centralbl. fur Chir.* No. 35) strikingly resemble those demonstrated by Prof Lister to the French Academy. Careful thermometric measurements have shown that the simple elevation of an extremity will be followed by a reduction of its temperature amounting to six or seven degrees. If in addition a moderate degree of cold be applied to the surface, "a contraction of the capillaries will ensue which may continue during several hours, the member all the while remaining pale and cold."

In attempting to utilize these results, which failed to impress us as original or even novel, Dr. Wolff employed in a number of operations on extremities the prolonged application of cold to the elevated limb and succeeded admirably. The procedure consists in freezing the part during from fifteen to thirty minutes before the operation by irrigation with cold water. During the operation the extremity should be held in a vertical position. By this means the loss of blood is surprisingly diminished, little operations being absolutely bloodless, and greater ones minus the danger of secondary hemorrhage, so frequent after constriction by Esmarch's bandage. The method under consideration has been applied in ten operations, and generally with success. In a patient aged ten years, Bardeleben performed Sime's amputation with but an insignificant hemorrhage. It is necessary to add, however, that the process of refrigeration was commenced several hours before the operation, and that during the latter the femoral artery was compressed. —*Lancet and Clinic.*

**Treatment of Diphtheria.**—Dr. D. D. Howell writes to the Lancet:

The late Mr. John Scott used to treat severe cases of cynanche with a "scavenger" viz. calomel, jalap, and scammony, after which they usually got well. Looking at a diphtheritic throat one day, I said to myself, Why should I not give this patient a scavenger? I did so, and remarked that the subsequent course of the disease was certainly cut short. I have since adopted the plan, and invariably found that the same good results have followed; the tendency to fresh deposits has much diminished, and in many cases is wholly prevented. I can call to mind more than one case in which the attempt at a fresh deposit was clear, but very feeble.

Unhappily I have seen a good deal of diphtheria at different times since its appearance in this country, about twenty-two years ago, and have treated it, on the whole, not unsuccessfully, but it was only a about a year ago that the above circumstances almost accidentally forced the conviction on my mind that *elimination of the poison* ought to be the first object in treatment.

How this elimination takes place through the bowels I know not, but diphtheria is a disease of blood-poisoning, and there is no reason why the poison should not be eliminated through the bowels, any less or more than in enteric fever—in throat fever than in bowel fever. I cannot refuse the evidence of my experience, that since I have adopted the practice of purging at the outset the course of the disease has been invariably more favorable, nor the testimony of those who declare that the dejections thus produced are abominably offensive. At any rate, the practical effect of the "scavenger" recommends itself to my mind in a much higher degree than the scientific inaction of temporizing and waiting till the disease has run its course, however masterly such inaction may appear; or than the inferior, but by no means futile plan, of trying to neutralize the effects of the poison. Invaluable as the scientific knowledge is, it ought not to stop short, nor rest content, without improvement in practice.

Elimination, then, first through the bowels, then through the kidneys. Chlorate of potash drinks should be given frequently, incessantly. Where the nose is affected, the same solution or that of permanganate of potash should be drawn up frequently through the nostrils, so as thoroughly to cleanse them. When the patient cannot swallow, nutrient enema should be given four times a day; not too frequently if they are to be retained. For this, nothing is better than Dr. Munk's mixture, a tumblerful of milk gruel, a new-laid egg, a tablespoonful of brandy.

**The Chlorine Mixture in Scarlet Fever and Diphtheria.**—In the lectures of Dr. Thomas Watson he recommends a solution of chlorate of potassa in water (a drachm to the pint), as a drink for patients in scarlet fever and in typhoid forms of continued fever. He says: "Chlorine has been strongly impressed upon my notice in several forms of scarlet fever."

In the fourth volume of the Medical Gazette Messrs. Taynton and Williams, of Bromley, write in high praise of this remedy.

The formula is: Two drachms of chlorate of potassa are to be dissolved in two ounces of hydrochloric acid, previously diluted with two ounces of distilled water. The solution must be put immediately into a stoppered bottle, and kept in a dark place. Two drachms of this solution, mixed with a pint of distilled water, constitute the chlorine mixture, of which a tablespoonful or two, according to the age of the patient, may be given for a dose, and frequently repeated.

In my own practice I have used this mixture for many years in cases of scarlet fever, and with considerable benefit to my patients, but I neglected it for a long time and used other remedies. Recently, however, my attention has again been called to its use in diphtheria. I have had considerable experience in this fearful form of disease. In one locality in one of the rural districts of our city, in a compass of about one square mile, I had nine severe cases of diphtheria, and other physicians had cases in the same locality. In my cases I used the chlorine mixture freely and with great benefit to my patients. Out of the nine, two died, of the most malignant type of the disease. One, a boy of nine years of age, was the worst case I ever saw. It was in the month of September, and the mosquitoes were very bad in the neighborhood. Wherever one of them bit the child the skin turned purple.

I have had considerable difficulty in procuring the chlorine mixture from the druggists, as I found none who kept the article already prepared, and when I would write for chlorate of potassa and muriatic acid I always procured a mixture as clear as water. My formula is :

| | | |
|---|---|---|
| ℞ | Potassa chloratis | ʒj |
| | Acidi muriatici | gtt. xl. |
| | Syrupus | f. ℥ ij. |
| | Aquæ | f. ℥ iv. M |

Dose according to age of patient.

This, when mixed, would invariably produce the clear white mixture spoken of above. The proper way is to put the chlorate of potassa into the bottle intended for the mixture, drop the acid upon it, and at once the chlorine will be liberated. Then add the sugar and water. The mixture, when prepared in this way, will have a deep straw color, and will smell strongly of chlorine.—Dr. Patterson, in *Med. and Surg. Rep.*

**Gelseminum for Hectic.**—The preparations of gelseminum have been steadily growing in favor for several years, and from an extended experience with them, partly upon theoretical and partly upon inferential grounds, I was led to try them in cases of hectic, which had resisted other well-known remedies until its pre-eminence has become so conclusive as to suggest its recommendation to the profession. Four years ago, in experimenting with it, and testing its action on the heart and pulse with the sphygmograph, I observed that the number of respirations was reduced before its toxic influence was manifested. Several deaths from overdoses, recorded in the journals, showed an action upon the respiratory centres similar to that of curare or woorara. Practical experience, moreover, with it, in small doses, has long shown its influence upon circulation and its sedative effect in certain neuralgias.

Finally, a writer in the Lancet for September of the current year collates the accounts of death from the drug in poisonous doses, and in the summing up shows that sedation and finally paralysis of the respiratory centres has been constantly present.

Now from these facts the inference has appeared a correet one that it should act favorably in the treatment of a respiratory affection characterized by irritation, and having its seat and origin in the pulmonary tissues. In a very large number of cases it has not failed, and without giving them in detail it will probably suffice to say that I have in prescribing it, even after the failure of favorite and well-known remedies, acquired confidence in it, and have found that in doses of two drops of fluid extract, or 10 to 12 of the tincture every two hours, it will, in most instances, within forty-eight hours arrest the chill, moderate the cough and allay the fever. The period of administration, however, is not always so short. It may be used continuously if necessary to maintain sedation, and without interference with other medicines or effect upon digestion or the excretions. It should be added that exceptions are likely to occur in cases with mesenteric complication and colliquative diarrhœa, and while not contra-indicated, it may sometimes disappoint expectations.—*New York Medical Record.*

**Potato Eating and Diphtheria.**—Dr. M. C. Keith, of Lincoln, Neb., writes to the Chicago Inter-Ocean, charging much of the diphtheria prevalent to eating white (Irish) potatoes. He says :

Some seventeen years ago the attention of my father, Dr. Alvan Keith, late of Augusta, Me., was called to the fact that children who were not fond of the tuber known as the Irish potatoes were not subject to attacks of that much-dreaded malady, diphtheria. Following out this hint, he advised families of his friends to avoid the use of this vegetable among the children, and until his decease he was accustomed to make the assertion that rotten potatoes produced the throat disease known as diphtheria.

In my practice in this city and county the offer has been to treat any one free of compensation if they would avoid the use of Irish potatoes. As a sequence not one of the patients who was not a potato eater has been threatened with the disease. In many of the inland towns of this State the writer has patients, and in some of the infected districts the families of those who have learned of this simple preventive have escaped any attack of throat disease, although the potato eaters on either side of them have unfortunately had cases of diphtheria which resulted fatally.

The writer rests his assertions upon a record of twelve years of his father's practice prior to 1861, and seventeen years of his own, covering a period of twenty-nine years, and includiug a personal knowledge of 1100 cases of diphtheria. The tuber known as the Irish potato is specified, since it is believed that sweet potatoes do not have the effect of producing any disturbance in the human animal economy. If these assertions are proven, the result must relieve the world from a disease much to be dreaded, and a malady which has caused many heartaches upon two continents.—*Med. Reporter.*

**Prolapse of the Rectum in Infants.**—In a recent number of the Wiener Medizinische Zeitung Dr. Basevi suggests an improved method of treating this troublesome affection, which he finds most successful.

He cauterizes the mucous membrane of the intestine lightly with nitrate of silver, and replaces the gut. Subsequently enema of tannin, alum and ice-water are ordered, together with very strict diet, with a view to prevent enteritis. Should these measures fail and the intestine continue to come down, he uses his bandage as follows : the child is held by two nurses, with its buttocks up, over the bed, one securing the upper portion of the body, the other the slightly abducted knees somewhat up in the air. This position is most favorable for the reduction of the prolapsed rectum, because the child cannot bear down. After reposition, the surgeon stands on the right side of the bed with the thumb of the left hand pressing the child's left buttock to the right, while the fingers bring the right buttock toward and against it. With the right hand several strips of plaster of some two finger-breadths are drawn from below upward and outward, overlapping one another, across the buttocks, from one trochanter to the other. The strips should approach the perineum as closely as possible. As a support to the plaster, a spica bandage of two or three finger-breadths is run over the lower part of the body. A gutta-percha or waxed paper covering can be used to keep the buttocks clean during defecation, and this bandage can be retained in position for a couple of weeks. If diarrhea be present, astringent enemata may be employed; if constipation, laxative enemata; and these should be given by the physician himself, for fear of disturbing the bandage, which can be changed without difficulty when necessary.—*Press and Cir.*

**Clinical Value of the Thermometer in Abdominal Affections.**—By Prof. Peter.—The physician may be called to a case of disease of the stomach; he finds no material proof of the existence of cancer, but he may feel intuitively that there is one. In such a case the application of the thermometer is of real importance. Being called in consultation by my distinguished confrere, M. Leudet (de Rouen) to the case of a man 52 years of age, percussion of the epigastrium, practiced *en dedans*, showed certain points of dullness. Under these circumstances, I had recourse to the thermometer to clear up the obscurity of the diagnosis. In the epigastric depression the thermometer registered 37.5° (99.4° Fahr.); but it is known that the normal temperature of the epigastrium in the healthy individual is about 35.5° (about 96° Fahr.). In painless atonic dyspepsias, the temperature remains unchanged. The result of my researches goes to show that the temperature in the vicinity of a cancerous lesion is elevated from 1° to 1.5° (centigrade) above the normal. This is not due to phlegmasia but to irritation. The fact is, in my opinion, significant. In simple gastralgia, there may be elevation of temperature, but it is ephemeral, and only lasts as long as the pain continues. This is a fact which appears to me to be worthy of remark, and which is interesting because it enables us to comprehend how, even in cancer, a cauterization of the epigastrium, resorted to opportunely, may dissipate the hyperemia; to say nothing of the important diagnostic deductions derivable from this means.—*La France Medicale.*

**Alkalies in Dyspepsia.**—Alkaline bicarbonates, being acid salts, if taken into the stomach before meals, when the mucous membrane is either neutral or alkaline, will be absorbed undecomposed into the blood and cause that increased acidity of the urine which has been observed.

But when taken during digestion, the acid contents of the stomach decompose them; carbolic acid is liberated, escaping by the mouth, "whilst the alkaline bases pass into the system and cause the urine to assume an alkaline reaction."

The therapeutic importance of Dr. Ralfe's observations are obvious; and I will give his conclusions in his own words.

1. In cases of acid dyspepsia arising from the excessive formation of acid within the system, as in lithamia, the alkaline bicarbonates should not be administered before food, but after.

2. The administration of alkaline bicarbonates before meals is indicated in those cases where the free acid is formed in the stomach itself, the result of fermentative changes of undigested food or morbid mucus, when it is necessary to diminish the too high degree of acidity thus caused, in order to permit digestion to be properly performed.—*Md. Med. Journal.*

**Treatment of Burns.**—Having submitted sundry cases of burns and scalds to the treatment by bicarbonate of soda, the following conclusions arrived at by the writer may be trusted as being reliable :

Points in its favor—

1. It is an exceedingly clean application.
2. The soothing effect is instantaneous.
3. For slight burns it is admirable.

Points against its general adoption—

1. The "soda" solution requires to be ice-cold, appearing to part with its soothing quality the instant its temperature is raised, as by immersion in it, so that the relief afforded is but momentary.
2. The quantity of bicarbonate required may be enormous, according to the extent of injury inflicted, nothing short of a saturated solution being of the slightest good.
3. Should the burn necessitate an unsparing resort to cold water, it may cause the blood to congest on the internal organs.
4. The prescriber must be well assured that he selects the bicarbonate, the ordinary carbonate Scotch soda, washing soda, or soda of the shops being far too irritant and caustic to be applied to a raw, highly sensitive surface.—Mr. Herbert Kiernander, in *London Lancet.*

**Intussusception in Infants.**—There is no absolute pathognomonic symptom of the disease, and it is difficult, particularly in the early period of the attack, to make the diagnosis. Intussusception may be confounded with acute indigestion, gastritis from poisoning, acute dyssentery, colic, cholera infantum and with other forms of internal strangulation of the intestine. The sudden development of abdominal pain in an infant above the age of three months, with persistent vomiting, soon followed by bloody stools and tenesmus, points very strongly to intussusception. If the presence of an abdominal tumor of recent occurrence can be ascertained, there can scarcely exist a doubt as to the special character of the disease. If, with the above described symptoms, a tumor can be felt in the rectum, a positive diagnosis can at once be made.

**Hints for Headaches.**—Dr. Day said, in a late lecture :

Whatever be the plan of treatment decided upon, *rest* is the fisrt principle to inculcate in every severe headache. Rest, which the busy man and the anxious mother cannot obtain so long as they can manage to keep about, is one of the first remedies for every headache, and we should never cease to enforce it. The brain, when excited, as much needs quiet and repose as a fractured limb or an inflamed eye, and it is obvious that the chances of shortening the seizure and arresting the pain will depend on our power to have this carried out effectually. It is a practical lesson to be kept steadily in view, in that there may lurk behind a simple headache some lesion of unknown magnitude which may remain stationary if quietude can be maintained.—*London Medical News.*

**The External Use of Digitalis in Suppression of Urine.**—Dr. P. C. Russell says in the British Medical Journal:

A married woman, aged 35, was attacked by acute albuminuria. The disease resisted the usual remedies. She became extremely œdematous, with congestion or œdema of both lungs. Respiration was rapid; the pulse weak and rapid. She became semi-comatose, and there was suppression of the urine for thirty-six hours. The case appeared hopeless; but having read in the British Medical Journal a case in which the external use of digitalis was effectual in restoring the secretion of urine, I determined to try it. I ordered half-an-ounce of the tincture on a large linseed-meal poultice, to be applied to the abdomen. Next day I was agreeably surprised to find her vastly improved, quite conscious and cheerful. The œdema was very much diminished. Respiration was easy, and the pulse nearly natural. I was informed that in one hour after the application a copious flow of urine commenced and continued all night, and, what was very remarkable, the urine, which the day before contained a large quantity of albumen, was now quite free from it. Convalescence was rapid, and she is now quite well.

**Silicylic Acid Enemata in Dysentery.**—Dr. Berthold employs an enema consisting of one gram of salicylic acid, three hundred grams of distilled water, and alcohol q. s.

In dysentery with tenesmus and bloody stools he administers it every four hours. The tenesmus diminishes, and the number of stools is rapidly reduced, the fecal matters gradually acquiring their normal appearance, the temperature diminishing, and the appetite returning.—*Union Medicale.*

**A Sure and Rapid Cure for Hiccough.**—Under this title Dr. Grellet, of Vichy, states that he has never failed in immediately relieving hiccough, *i. e.* not dependent upon any appreciable morbid condition, by adminstering a lump of sugar imbibed with vinegar.—*Revue Medicale.*

**Salicylate of Soda in Rheumatism with High Fever.**—Salicylate of soda need rarely be given in larger doses than from ʒi to ʒiii daily in order to produce all its best effects, which will be marked in one or two or three days at most; and then the dose should be gradually diminished until some time after complete convalescence has set in.—*Md. Med. Journal.*

**Bromide of Potassium in Coughs.**—Dr. Fothergill gives it to relieve various forms of cough. In that of phthisis he gives hydrobromic acid mixed with spirits of chloroform, in preference to any cough mixture; having a wholesome disgust for the long list under that category. He is very much opposed to the use of chloral in such cases, from the dangerous habit it incurs.—*Md. Med. Journal.*

**Guaco in Cancer.**—There seems no reason *a priori* why some internal remedy may not be discovered which shall do for epithelial and other new growths what iodine does for syphilitic new formations. Dr. Schmidt suggests guaco, which he has used in the form of tincture and plaster.—*Times.*

